# Excessive Treadmill Training Enhances Brain-Specific MicroRNA-34a in the Mouse Hippocampus

**DOI:** 10.3389/fnmol.2020.00007

**Published:** 2020-01-30

**Authors:** Lin Xu, Yi Li Zheng, Xin Yin, Sheng Jia Xu, Dong Tian, Chen Yu Zhang, Sen Wang, Ji Zheng Ma

**Affiliations:** ^1^Department of Exercise and Health, Nanjing Sport Institute, Nanjing, China; ^2^The Research Center of Military Exercise Science, The Army Engineering University of PLA, Nanjing, China; ^3^State Key Laboratory of Pharmaceutical Biotechnology, Collaborative Innovation Center of Chemistry for Life Sciences, Jiangsu Engineering Research Center for MicroRNA Biology and Biotechnology, NJU Advanced Institute for Life Sciences (NAILS), School of Life Sciences, Nanjing University, Nanjing, China; ^4^Department of Geriatric Cardiology, First Affiliated Hospital of Nanjing Medical University, Nanjing, China

**Keywords:** treadmill training, hippocampus, miRNAs, overtraining, normal training

## Abstract

**Background**: An imbalance between total training load and total recovery may cause overtraining (OT). The purpose of the present study was to verify the effects of OT on the expression of brain-derived neurotrophic factor (BDNF), its receptor tropomyosin receptor kinase B (TrkB) and p75 and the dynamic expression patterns of brain-specific miR-34a and miR-124 or inflammation-related miR-21 and miR-132 in the mouse hippocampus.

**Method**: Eight weeks old C57BL/6J mice were randomly assigned to the control (CON), normal training (NT) and OT groups. An 8-week OT training protocol was applied to evaluate the phenotype of mice endurance (incremental load test, ILT) and cognitive capacity (Morris water maze test). We used qRT-PCR and immunoblotting to detect changes in the molecular level of hippocampal samples.

**Result**: Compared with the CON, both NT and OT decreased bodyweight after 8-week training. After 8-week of training, NT increased the exhaustion velocity (EV) while the EV of OT was lower than NT. Mice in NT decreased the escape latency than CON. The percentage of time spent in the probe quadrant and the number of crossing platform times in NT were higher than CON and OT. The BDNF, p75 and TrkB mRNA levels were increased in NT than CON, only the p75 mRNA was increased in OT. The NT exhibited increased protein levels of BDNF and TrkB compared to CON. The protein expression of BDNF was decreased in OT than NT and CON. The protein level of p75 in the OT was higher than in NT and CON. In addition, the phosphorylation level of TrkB in OT was higher than CON and NT. Only the miR-34a level was increased in the OT. Moreover, the expression of miR-34a was found to be negatively correlated with the expression of BDNF, and the increase in miR-34a level was accompanied by a decrease in performance.

**Conclusion**: In summary, the training-evoked increase in the BDNF level may help to improve performance, whereas this conditioning is lost after OT. Moreover, miR-34a potentially mediated changes in the expression of BDNF and may reflect the decrease in performance after OT.

## Introduction

Exercising regularly is a potent force of nature with significant potential for maintaining body health throughout the lifespan. Aerobic exercise modifies many molecular, physiological and structural changes in humans and animals, particularly in the hippocampus, the brain region critically important for memory consolidation and learning (Hamilton and Rhodes, 2015). Thus, exercise training has been recommended as a promising therapeutic strategy for countering age-related changes in the hippocampus, such as alterations in relational memory and mnemonic discrimination (Voss et al., 2019). For example, it has been shown that moderate exercise increases the size of the hippocampus in humans, which is also linked to enhanced memory (Erickson et al., 2011).

Neurotrophins are a family of closely related secreted proteins that play an important role in promote nervous development and neurite outgrowth (Levi-Montalcini, 1987). Among them, brain-derived neurotrophic factor (BDNF) is highly expressed in the brain, interacts with its receptor tyrosine kinase TrkB and p75 to regulate nervous system function like neuronal differentiation and survival, dendritic pruning, the patterning of innervation, synaptic function and plasticity in the central and the peripheral nervous system (Skaper, 2018). Activation of Trkb by BDNF can regulate the induction of hippocampal long-term potentiation (LTP) which is an important mechanism of hippocampal learning and memory (Kovalchuk et al., 2002). The p75 is a member of the TNF receptor superfamily that can bind to BDNF and transmits signals important for determining which neurons survive during development (Huang and Reichardt, 2001). At present, the precise molecular mechanisms regarding exercise-mediated neurogenesis are still not fully known (Baptista and Andrade, 2018; Cooper et al., 2018). Importantly, BDNF is a candidate mechanism underlying these exercise-induced benefits that help optimize brain plasticity outcomes *via* exercise intervention (Baptista and Andrade, 2018; Cooper et al., 2018). Acute high-intensity exercise increases the expression of BDNF (Venezia et al., 2017). Furthermore, MicroRNAs (miRNAs), small non-coding regulatory RNAs, are important regulators of various cellular processes *via* several signaling pathways relevant to exercise adaptation (Domańska-Senderowska et al., 2019). Moreover, exercise-induced memory improvements are accompanied by changes in the hippocampal miRNA-mRNA regulatory network (Fernandes et al., 2018). In addition, several specific miRNAs (brain-specific miR-34a; Agostini et al., 2011), miR-124 (Sun et al., 2015), inflammation-related miR-21 (Slota and Booth, 2019), miR-132 (Dong et al., 2018) are involved in the regulation of biological processes within the brain, including development, proliferation and apoptosis.

High training loads are generally used to improve sports performance during periodization phases. Emerging evidence has indicated that recovery periods are important for athletes to achieve an overcompensation period and to improve their performance, and inappropriate load management is a significant risk factor for acute illness and overtraining (OT) syndrome (Meeusen et al., 2013). This process has been defined as functional overreaching (FOR), nonfunctional overreaching (NFOR) and overtraining syndrome (OTS; Meeusen et al., 2013). At present, as OTS is a diagnosis of exclusion, it is important to screen for inflammatory, metabolic, hormonal, psychiatric, and infectious conditions that may be the primary reason for the decrease in performance (Meeusen et al., 2013). It is well established that mice subjected to a downhill running-based OT protocol induces the NFOR state (Pereira et al., 2012, 2014), which is defined as a performance decrement that may be reversed after weeks or months of recovery (Meeusen et al., 2013). This protocol was used by our research group. Based on the important role of BDNF and specific miRNAs in sustaining the physiological processes of the brain, we verified the effects of OT on BDNF/TrkB signaling and the dynamic expression patterns of brain-specific miR-34a and miR-124 and inflammation-related miR-21 and miR-132 in the mouse hippocampus.

We hypothesized that OT affects these key molecules, which may be useful for monitoring OTS and better understanding the negative adaptations that contribute to performance decrease.

## Materials and Methods

### Experimental Animals

A total of 90 male C57BL/6J mice (8 weeks old) were ordered from the Experimental of Beijing Vital River Laboratory Animal Technology (Beijing, China) and every five mice were housed in the same cages with a controlled temperature (22 ± 2°C). The mice were kept on a 12 h light/dark schedule (light: 9 AM to 9 PM, dark: 9 PM to 9 AM) and provided access to food and water. All mouse breeding and experimental procedures were approved by the Nanjing University Animal Care and Use Committee (ID: GPTAP014).

**Figure 1:**
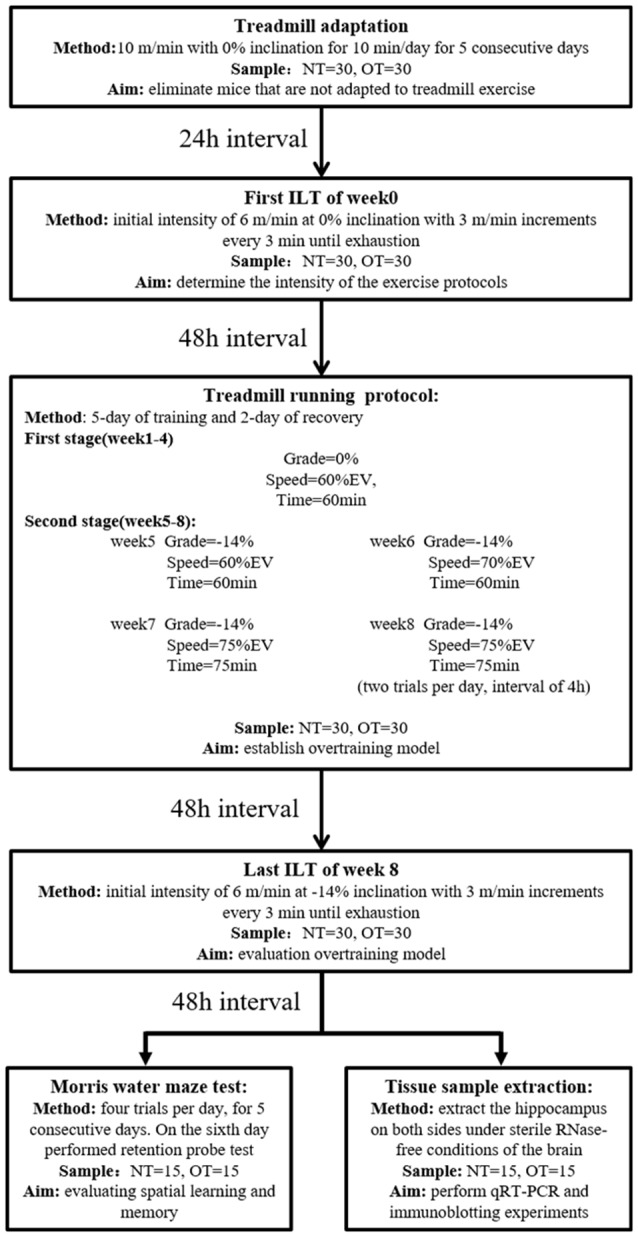
Experimental design. Mice (normal training, NT: *n* = 30, overtraining, OT: *n* = 30) exercised according to the training program, including a treadmill training protocol and two incremental load tests (ILT). During the second stage of the treadmill running protocol, the NT group ran at Grade = −14%, Speed = 60% EV and Time = 60 min. Forty-eight hours after the end of the last ILT, the mice were killed to take tissue samples (NT: *n* = 15, OT: *n* = 15) or perform Morris water maze experiments (NT: *n* = 15, OT: *n* = 15), respectively.

The rodents were randomly divided into three groups: the control group (CON; sedentary mice; *n* = 30), normal training group (NT; trained with adequate recovery, *n* = 30), the group overtrained by downhill running (OT; performed the OT protocol based on downhill running; *n* = 30). The experimental groups were trained or tested in an appropriately lit room between 1 PM and 5 PM (Figure 1).

### Acclimatization and Incremental Load Test (ILT)

As previously described (Pereira et al., 2012), the NT and OT groups were first acclimated to treadmill running (ZH-PT, Zhenghua, Anhui, China) at 10 m/min with 0% inclination for 10 min per day for a week (includes 5-day of treadmill adaptation, 1-day of ILT test, and 1-day of rest). Then, rodents performed the ILT with an initial intensity of 6 m/min at 0% inclination with 3 m/min increments every 3 min until exhaustion, which was defined as a mouse touching the end of the treadmill five times in 1 min. The mice were encouraged using physical prodding, and if a mouse became exhausted without completing the stage, the exhaustion velocity (EV = V + (n/b)*a, with V being the completed maximum speed, n is the duration in the incomplete stage, b being the duration of the stage, and a being the test increment) was calculated (Kuipers et al., 1985). The EV of each mouse was used to determine the intensity of the NT and OT protocols.

### Running OT Protocol and Performance Evaluation

Briefly, each experimental week of the 8-week running protocol consisted of 5 days of training followed by 2 days of recovery. The mice were first adapted to treadmill running for 5 days, and the exhaustion velocity (EV: m*min^−1^) of each mouse was used to determine the intensity of the exercise protocols. The CON group did not perform any exercise. During the first 4 weeks (i.e., the first stage) of the exercise protocol, the NT mice ran at a grade of 0%, the intensity was maintained at 60% of EV and the duration was 60 min per day in the 4th week. From the 5th week to the 8th week of the exercise protocol, the intensity and duration were maintained, but the rodents ran at a grade of −14%. The OT protocol was the same as the NT protocol during the first stage and the 5th week. However, in the 6th week of the OT protocol, the intensity was increased to 70% of the EV. In the 7th week of the OT protocol, the intensity and duration were increased to 75% of the EV and 75 min, respectively. In the 8th week of the OT protocol, the number of daily sessions was doubled than the 7th week. The rest interval between daily sessions during the 8th week was 4 h (da Rocha et al., 2015; Pereira et al., 2015b). Forty-eight hours after the end of the last training session in week 8, the NT group and the OT group performed the ILT in downhill running.

### Morris Water Maze Test

Spatial learning and memory performance of mice was evaluated by the Morris water maze test. The Morris water maze protocol has been described in detail in Morris’s previous report (Morris, 1984). In brief, the mice were placed in opaque water of a circular swimming pool and trained to find a platform hidden below the water surface in target quadrants according to the spatial cues in the experimental room. During the training to find the hidden platform, the mice were allowed to swim for a maximum of 60 s. One block of four trials per day (1 min swimming time, inter-trial interval of 40 min) was performed for five consecutive days. On the 6th day, mice were given a 60-s retention probe test during which the platform was removed from the pool. Data were collected using a ceiling-mounted video camera, and analysis by MobileDatum software (RD1101-MWM-G, MobileDatum, Shanghai, China). All Morris water maze tests were performed between 1 AM and 5 AM, and the water temperature is maintained at 20 ± 2°C.

### Hippocampus Extraction and Immunoblotting Analysis

The mice were sacrificed 48 h after the last ILT test to eliminate the effects of acute exercise stress. The hippocampus of each hemisphere was surgically excised under sterile RNase-free conditions, snap-frozen in liquid nitrogen and stored at −80 °C until use.

The hippocampi were processed with a Tissuelyser machine (Jingxin, Shanghai, China). The samples were lysed in RIPA buffer and protease inhibitor cocktail, and the protein supernatants were collected after centrifugation. The protein concentration was calculated using the BCA Protein Assay Kit (Thermo Scientific, Rosemont, IL, USA). Equal amounts of protein were loaded on 12.5% SDS-polyacrylamide gels, separated using electrophoresis, and then transferred to a PVDF membrane (Bio-Rad, Irvine, CA, USA). The membrane was blocked with 5% skim milk powder in TBST for 1 h and incubated with primary antibodies (phosphorylation-TrkB Y705, 1:1,000, Abcam, Cambridge, UK; TrkB, 1:1,000, Cell Signaling, Danvers, MA, USA; p75NTR, 1:1,000, Cell Signaling, Danvers, MA, USA; BDNF, 1:1,000, Proteintech Group, Inc., Rosemont, IL, USA; β-actin, 1:1,000, Proteintech Group, Inc., Rosemont, IL, USA) at 4°C overnight. The membrane was washed in TBST four times for 10 mins each, and the membrane was incubated for 1 h with anti-mouse IgG (1:10,000, Invitrogen, Carlsbad, CA, USA) or anti-rabbit IgG (1:5,000, Abcam, Cambridge, UK). Thereafter, the membrane was washed four times for 10 min. The bands were detected with the SuperSignal West Pico chemiluminescence substrate (Thermo Scientific, Rosemont, IL, USA).

### RNA Isolation

For RNA isolation, the frozen hippocampus was homogenized using TRIzol reagent (Thermo Scientific, Rosemont, IL, USA) according to the manufacturer’s protocol. The RNA quality and quantity were measured using a Nanodrop spectrophotometer (ND-1000, Thermo Scientific, Rosemont, IL, USA).

### Quantitative Real-Time PCR (qRT-PCR)

For miRNA expression analysis, reverse transcription was performed using miRNA-specific probes to generate corresponding cDNA products. qRT-PCR was carried out with a TaqMan PCR kit on a Roche LightCycler 480 ∥ sequence detection system. The real-time PCR conditions consisted of a predenaturation step at 95°C for 5 min, followed by 40 cycles of 95°C for 15 s and 60°C for 1 min. The following TaqMan^®^ miRNA assays used were: hsa-miR-132 (ID: 000457), hsa-miR-34a-5p (ID: 000426), hsa-miR-124-3p (ID: 001182) and hsa-miR-21 (ID: 000397). All reactions, including those for the reference controls, were run in duplicate, and the Ct values were calculated. The Ct values of the miRNAs were normalized to that of U6 snRNA, and the relative levels of miRNAs were determined using the formula 2^−ΔΔCT^.

For mRNA expression analysis, the oligo (dT) method (TaKaRa, Dalian, China) was used for the reverse transcription of protein gene mRNA to generate cDNA. qRT-PCR was then performed using SYBR Green dye (Invitrogen) and specific primers for BDNF, p75 and TrkB. The specific primers were designed using the NCBI primer design tool (Table 1). The Ct values were determined by setting a fixed threshold. The relative mRNA levels were normalized to that of β-actin using the 2^−ΔΔCT^ method as described above.

**Table 1:** The sequences of the primers used for amplification.

	Forward primer	Reverse primer
BDNF	5′-TCATACTTCGGTTGCATGAAGG-3′	5′-AGACCTCTCGAACCTGCCC-3′
p75	5′-CTAGGGGTGTCCTTTGGAGGT-3′	5′-CAGGGTTCACACACGGTCT-3′
TrkB	5′-CTGGGGCTTATGCCTGCTG-3′	5′-AGGCTCAGTACACCAAATCCTA-3′

### Statistical Analyses

All statistical analyses were conducted using GraphPad Prism 5 software. The values are expressed as the means ± standard errors of the means (SEM). According to Shapiro–Wilkes W-test, the data were normally distributed, and the homogeneity of the variances was confirmed by Levene’s test. Therefore, one-way analysis (ANOVA) of variance was used to examine the results of qRT-PCR. When one-way ANOVA indicated statistical significance, Bonferroni’s *post hoc* test was performed. The significance level was predetermined to be *p* < 0.05 unless otherwise indicated.

## Results

### Bodyweight and Incremental Load Test

**Figure 2:**
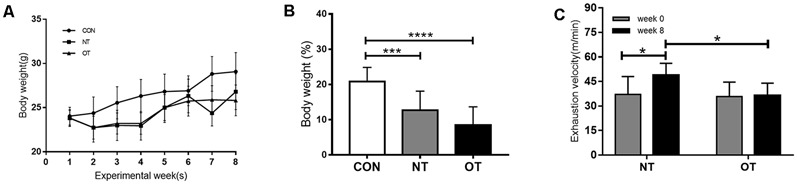
Bodyweight (g) responses in the experimental groups during the experimental weeks **(A)**. The percentage of body weight change between week 0 and week 8 in the experimental groups **(B)**. The effects of NT and OT group on the exhaustion velocity (EV), as determined during the ILT during weeks 0 and 8 **(C)**. The data represent the means ± SE of *n* = 15. CON: sedentary mice; NT: mice trained with adequate recovery; OT: mice that underwent the downhill running OT protocol. *Statistical significance (*P* < 0.05); ***statistical significance (*P* < 0.001); ****statistical significance (*P* < 0.0001).

Figure 2A shows the changes in body weight (g) in the experimental groups during the experimental weeks. As indicated in Figure 2B, bodyweight from week 0 to week 8 was significantly lower for the OT group (8.43 ± 5.25%, *p* < 0.0001) and NT group (12.68 ± 5.43%, *p* < 0.001) compared with the CON group (20.79 ± 4.06%). In addition, it is important to note that the bodyweight of the OT group tended to be lower than that of NT, although the difference failed to reach statistical significance.

As shown in Figure 2C, significant differences in EV were not observed between the NT group (37.13 ± 10.91) and the OT group (35.69 ± 9.01) during week 0. In addition, the EV of the NT group (48.88 ± 7.28) was significantly greater than that of the OT group (36.3 ± 7.67) during week 8. Compared with that during week 0, the EV of the NT group (*p* < 0.05) increased significantly during week 8, while there was no significant difference in the EV of the OT group (*p* > 0.05).

### Morris Water Maze Performance

**Figure 3:**
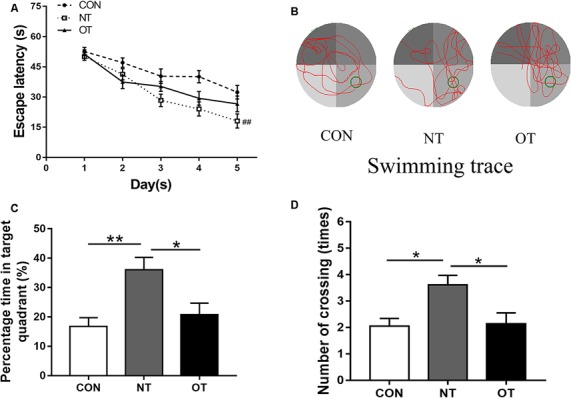
The Morris water maze test results in the three experimental groups. The escape latency of the experimental groups **(A)**. Representative samples of swimming trace during the probe test phase on day 6 **(B)**. The percentage time in target quadrant **(C)** and the number of crossing **(D)** during probe testing. The data represent the means ± SE of *n* = 15. CON: sedentary mice; NT: mice trained with adequate recovery; OT: mice that underwent the downhill running OT protocol. *Statistical significance (*P* < 0.05); **statistical significance (*P* < 0.01); ^##^statistical significance (*P* < 0.01) compared with the CON group on the 4th and 5th day.

The results showed that the escape latency of the three experimental groups in the first 3 days was similar and there was no significant difference in trial training (Figure 3A). NT group (29.35 ± 14.35) significantly (*P* < 0.01) decreased the escape latency by day 4 as compared to CON group (38.93 ± 14.43) and remained significant on days 5 (NT:26.57 ± 15.52, CON:32.36 ± 14.77, *P* < 0.01). From the 4th day to the fifth day, the NT group showed a lower escape latency than the OT group, but the difference was not significant (*P* > 0.05). Figures 3C,D shows the probe trial results, we found that the NT group (36.07 ± 15.20%) showed a significant increase in the percentage of time spent in the probe quadrant than CON group (16.75 ± 11.14%, *P* < 0.01) and OT group (20.72 ± 13.73%, *P* < 0.05). Similarly, we found that the number of times in the NT group (3.61 ± 1.50) crossed the original platform position was significantly increased compared to the CON (2.05 ± 1.36, *P* < 0.05) and OT groups (2.14 ± 1.83, *P* < 0.05). Representative swimming traces of the three experimental groups during the probe test are shown in Figure 3B.

### Expression of mRNA

**Figure 4:**
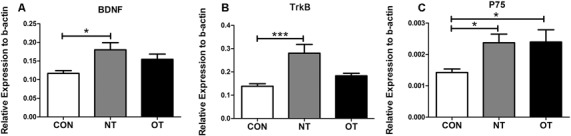
The mRNA expression in the hippocampus in the three experimental groups. brain-derived neurotrophic factor (BDNF; **A)**, TrkB **(B)**, p75 **(C)**. The data represent the means ± SE of *n* = 15. CON: sedentary mice; NT: mice trained with adequate recovery; OT: mice that underwent the downhill running OT protocol. *Statistical significance (*P* < 0.05); ***statistical significance (*P* < 0.001).

As shown in Figure 4A, we measured the mRNA expression levels in the hippocampus in the three experimental groups. The mRNA expression levels of BDNF were significantly higher in the NT group than in the CON group (*p* < 0.05). Compared with that in the NT group, the excessive training program reduced BDNF expression in the OT group, but the mRNA expression in the OT group was still higher than that in the CON group. The relative mRNA expression of TrkB among the different groups is shown in Figure 4B. The analysis of the mRNA expression data showed that TrkB mRNA level in the NT group was significantly higher than those in the CON group (*p* < 0.001). There was a rise in the TrkB mRNA level in the OT group compared with the CON group after 8 weeks of excessive training, but there was no significant difference. The relative mRNA expression of p75 among the different groups is shown in Figure 4C. According to the data, the mRNA expression of p75 was significantly increased in the NT and OT groups compared with the CON group (*p* < 0.05).

### Protein Expression and Phosphorylation Level in the Hippocampus

**Figure 5:**
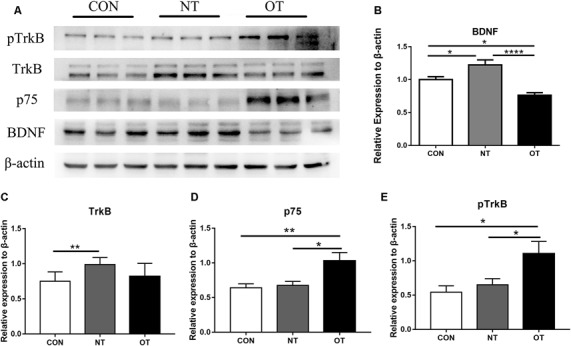
The representative protein expression and phosphorylation level results of the three experimental groups are shown in figure **(A)**. Protein levels (arbitrary units) of BDNF **(B)**, TrkB **(C)**, p75 **(D)** and the respective β-actin controls in the hippocampus. pTrkB **(E)** shows the phosphorylation level of TrkB. The data represent the means ± SE of *n* = 9. CON: sedentary mice; NT: mice trained with adequate recovery; OT: mice that underwent the downhill running OT protocol. *Statistical significance (*P* < 0.05), **statistical significance (*P* < 0.01), ****statistical significance (*P* < 0.0001).

Figure 5A shows that the protein expression and phosphorylation levels in hippocampal tissue were different among the three experimental groups. The relative expression of BDNF (*p* < 0.05) and TrkB (*p* < 0.01) protein in the NT group was significantly higher than that in the CON group (Figures 5B,C). The protein expression of p75 was higher in the NT group than in the CON group, but the difference was not significant (*p* > 0.05; Figure 5D). The BDNF (*p* < 0.05) protein expression level in the OT group were significantly decreased compared with those in the CON group (Figure 5B). Compared with the CON group, the p75 (*p* < 0.01) protein expression level in OT group was significantly increased, while the TrkB protein expression was also increased but not significantly (Figures 5C,D). In addition, the relative protein expression of BDNF (*p* < 0.0001) and TrkB (*p* > 0.05) was lower in the OT group than in the NT group, which significant only in BDNF (Figures 5B,C). In contrast, the protein expression of p75 (*p* < 0.05) in the OT group was significantly higher than that in the NT group (Figure 5D). Figure 5E shows that the phosphorylation level of TrkB in the OT groups was significant increase than in CON (*p* < 0.05) group and NT (*p* < 0.05) group. The phosphorylation of TrkB in the NT group was higher than that in the CON group, but the difference was not significant (*p* > 0.05). These results suggest that those protein and phosphorylation level are differentially expressed in the hippocampus as a result of different exercise protocols.

### The Relative Levels of miRNAs in the Hippocampus

**Figure 6:**
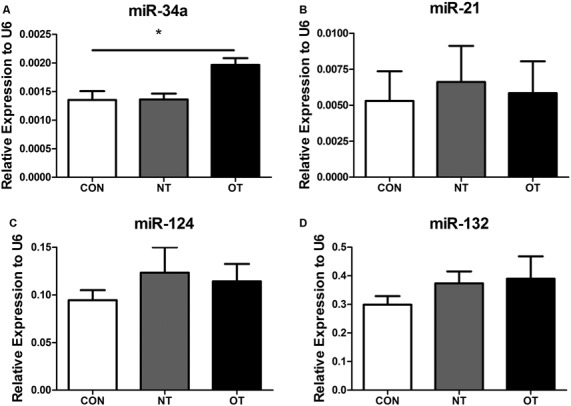
Expression of miR-34a **(A)**, miR-21 **(B)**, miR-124 **(C)** and miR-132 **(D)** in the hippocampus of the three experimental groups. The data represent to means ± SE of *n* = 15. CON: sedentary mice; NT: mice trained with adequate recovery; OT: mice that underwent the downhill running OT protocol. *Statistical significance (*P* < 0.05).

As shown in Figure 6A, we examined the expression of miR-34a in hippocampal tissue after excessive training by qRT-PCR. The data showed that the expression level of miR-34a was significantly upregulated in the OT group compared with the CON group (*p* < 0.05). However, similar results were not obtained by additional qRT-PCR analysis. Figures 6B–D show the results of quantitative RT-PCR demonstrated that the expression levels of miR-21, -124, and -132 in the NT and OT groups were higher than those in the CON group, although the differences failed to reach statistical significance.

**Figure 7:**
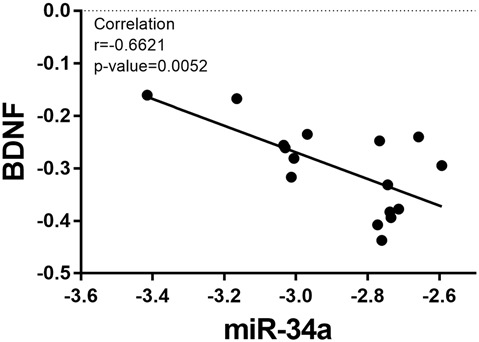
Correlation between miR-34a expression and BDNF expression. A direct negative correlation was observed between miR-34a expression and the protein levels of BDNF in hippocampal tissue (*r* = −0.6621, *p* = 0.0052).

Based on the fact that the protein expression of BDNF decreased in the OT group, the relative expression of miR-34a increased. We analyzed the correlation between the expression of miR-34a and the relative protein expression of BDNF after 8 weeks of excessive training. As indicated in Figure 7, the data showed that miR-34a was moderately negatively correlated with BDNF protein level in hippocampal tissue (*r* = −0.6621, *p* < 0.01).

## Discussion

The time frame of training and recovery may be important parameters for performance gain and loss. Overall, the above findings suggest that the OT protocol-induced increase in the miR-34a level may be linked to a maladaptation condition; thus, miR-34a may represent a distinction between adaptation and maladaptation.

It is well established that the BDNF/TrkB signaling system is one of the major systems involved in exercise-mediated hippocampal neurogenesis in response to acute exercise and adaptation to chronic exercise (Chou et al., 2018; Firth et al., 2018; Liu and Nusslock, 2018; Hill and Polk, 2019). Many mechanisms are involved in such adaptation, exerting significant regulatory control over many facets of a neuronal function and leading to the biological effects of the hippocampus, including cognitive functioning and neurogenesis (Chou et al., 2018; Firth et al., 2018; Liu and Nusslock, 2018; Hill and Polk, 2019). A previous study has shown that treadmill exercise in mice increases BDNF expression by regulating BDNF mRNA and protein expression in the hippocampus (Fahimi et al., 2017). Similar changes were observed in the NT group in the present study. BDNF and TrkB, as a BDNF-specific receptor, are equally functionally dependent upon on one another (Skaper, 2018), and the expression of TrkB has also been found to be acutely increased in trained mice (Liu et al., 2008). Moreover, the TrkB protein expression level is positively correlated with passive avoidance performance (Liu et al., 2008). Our study also showed that the TrkB mRNA and protein levels in the NT group increased in the present study. However, these positive changes were not found in the OT group. Moreover, autophosphorylation of the TrkB catalytic domain at tyrosine 705 is considered a critical step in TrkB receptor activation, which followed by activation of various signaling pathways (Huang and Reichardt, 2003). The phosphorylation of TrkB at Y705 only increased in the OT group. In addition, the mRNA level of p75, a tumor necrosis factor receptor (Skaper, 2018), increased both in the NT and OT groups. However, only the protein level of p75 increased in OT groups.

Furthermore, in animal models, chronic exercise training robustly increases the expression of BDNF and improves memory performance; there is also reasonable evidence to suggest that BDNF may mediate the exercise-memory interaction (Loprinzi, 2019). Similarly, our study also showed these changes. Moreover, the transmembrane domain of the p75 stimulates phosphorylation of the TrkB during brain injury, Alzheimer’s disease, and epilepsy (Saadipour et al., 2017). Elevated p75 protein/TrkB phosphorylation levels were also found in OT groups in our study, which likely indicated maladaptation. Taken together, our results suggest that NT mainly activates adaptive BDNF/TrkB signaling, while OT likely involved p75 and TrkB phosphorylation. It is known that the performance decrease induced by excessive training is a hallmark of an NFOR/OT state (Meeusen et al., 2013). However, given that some alterations found in OTS/NFOR/FOR may result from overload training regardless of the performance state, alterations may not always differentiate between OTS, NFOR and FOR but instead indicate excess training.

Recently, it has been suggested that altered miRNA profiles following exercise may be useful biomarkers of health and adaptation for intervention strategies (Domańska-Senderowska et al., 2019). Thus, monitoring the differential expression of miRNAs related to molecular patterns of communication triggered during/after exercise as a response may better elucidate the recovery and adaptation/maladaptation responses to the training load. Brain-specific miR-34a and miR-124 are recognized as regulators of signaling pathways relevant to neurophysiology and neuropathology (Agostini et al., 2011; Sun et al., 2015). miR-34a, as a tumor suppressor transcript, is abundantly expressed in the adult mammalian brain (Agostini et al., 2011). Previous work has suggested that the pre-inhibition or suppression of miR-34a improves neuronal survival in the presence of a variety of neurotoxins implicated in Parkinson’s disease (Horst et al., 2017). Its elevation may also play a role in neuronal demise in animal models of Alzheimer’s disease, and its suppression may be generally neuroprotective (Horst et al., 2017). Moreover, swimming intervention can delay d-galactose-induced brain aging in rats *via* suppressing miR-34a-mediated autophagy impairment and abnormal mitochondrial dynamics (Kou et al., 2017). However, an increase in miR-34a level in the OT group was observed in the present study, indicating that excessive training may activate these maladaptation consequences. In addition, miR-124 is also the most abundant miRNA in the brain (Sun et al., 2015). Furthermore, the aberrant expression of miR-124 has been found to contribute to pathological conditions involving the central nervous system (Sun et al., 2015). A previous study also suggested that exercise exerts a positive impact on stress resilience in singly-housed mice that may be mediated by decreased miR-124 expression and increased Nr3c1 expression in the hippocampus (Pan-Vazquez et al., 2015).

In addition, because muscle damage, inflammation, and different training phase responses may normally occur during exercise training or stress from OT designed to optimize performance (Meeusen et al., 2013; Pereira et al., 2014; da Rocha et al., 2017), it is critical to contextualize the assessment of inflammation-related miRNA with other assays concurrently. miR-132 has also been shown to be enriched in the mammalian brain and has been found to be highly conserved and enriched in neurons (Cheng et al., 2007). miR-132 has also been reported to participate in toll-like receptors responses and potentiates anti-inflammatory signaling (Shaked et al., 2009). In mammalian neurons, when miR-21 is aberrantly expressed, it contributes to inflammatory disease and has proinflammatory effects (Sheedy, 2015). A previous study showed that voluntary exercise suppresses miR-132 expression in the hippocampus of SAMP8 mice, which is associated with cognitive improvement (Dong et al., 2018). In addition, both exercise and tamoxifen have synergistic effects in reducing the expression of miR-21 in an estrogen receptor-positive breast cancer model to reduce mammary tumor burden in mice (Khori et al., 2015). These studies suggest that exercise may have positive effects on pathological conditions through the miR-132/miR-21 pathway. However, these changes have not been found in the NT group in normal mice. In addition, OT did not affect these two miRNAs in the present study.

Taken together, our study showed that with regard to the selected miRNA, only miR-34a was responsive to OT. More importantly, the increase in miR-34a level in the hippocampus in mice was accompanied by a decrease in motor and memory performance, and be negatively correlated with the expression of BDNF, which plays an essential role in exercise-induced neuroplasticity (Szuhany et al., 2015), likely indicating that miR-34a may be involved in the maladaptation consequence of OT. Training-induced phenotypic maladaptation is a consequence of repetitive stimulation from individual exercise bouts. In addition, the body weight in the OT group was unchanged in our study, although the low body weight gain and food intake were related to exhaustive training and OT (Pereira et al., 2015a). Thus, the precise roles and activity of miR-34a in the hippocampus, which is modulated by factors that control the expression of miR-34a, as well as those of its downstream target genes and signaling pathways, should be further investigated in relation to the OT-response continuum.

## Conclusion

Together, our findings of miR-34a-mediated BDNF changes may provide new insight into the maladaptation physiological responses to exercise and potentially make miR-34a a viable target for monitoring OT.

## Data Availability Statement

All data generated during the current study are available from the corresponding author upon reasonable request.

## Ethics Statement

The procedures for care and use of animals strictly followed the Guide for the Care and Use of Laboratory Animals published by the US National Institutes of Health (NIH Publication No. 85-23, revised 1996). The protocol was approved by the Nanjing University Animal Care and Use Committee.

## Author Contributions

LX, CZ, and JM designed the research. LX, YZ, and SW performed the experiments, analyzed the data, and revised the final manuscript with graveness. YZ, SX, DT, and XY helped with sample collection, laboratory materials, and reagents. All authors approved the final publication of this manuscript.

## Conflict of Interest

The authors declare that the research was conducted in the absence of any commercial or financial relationships that could be construed as a potential conflict of interest.
